# Neuroinvasive West Nile Virus (WNV) Encephalitis With Anton Syndrome: Epidemiology and Pathophysiology Review

**DOI:** 10.7759/cureus.26264

**Published:** 2022-06-23

**Authors:** Bahadar S Srichawla

**Affiliations:** 1 Department of Neurology, UMass Chan Medical School, Worcester, USA

**Keywords:** leukoencephalopathy, neuroinfectious disease, flavivirus, anton–babinski syndrome, anton's syndrome, west nile virus, neuroinvasive west nile virus, internal medicine, infectious disease, neurology

## Abstract

The West Nile virus (WNV) belongs to the genus of flaviviruses and is known to cause irreversible neurologic deficits. Neuroinvasive WNV cases continue to be rare and have a higher prevalence in South America, Africa, and Asia. Here we report a 55-year-old female from North America who presented with acute-onset encephalopathy, fever, myalgias, and Anton syndrome. Neuroradiographic findings included diffuse white matter abnormalities of both cortical and subcortical structures and the patient was diagnosed with posterior reversible encephalopathy syndrome (PRES). Further workup revealed WNV antibodies in both cerebrospinal fluid (CSF) and serum. Management of WNV encephalitis continues to be poor and thus the patient was referred to a long-term care facility. Furthermore, Anton syndrome is a rare focal neurologic deficit that has never been previously associated with the WNV. This case aims to highlight the epidemiology of WNV in the United States, the mechanisms of WNV encephalitis, and an overview of Anton syndrome.

## Introduction

The Flaviviridae family of viruses are classified as single-stranded ribonucleic acid (RNA) viruses that are enveloped. The classic vector of the flaviviruses are arthropods (mosquitos and ticks). Therefore, these viruses are also referred to as arboviruses. Well-known viruses that belong to this family include the Dengue virus, Zika virus, West Nile virus (WNV), and Powassan virus, among others. WNV was first identified in Uganda, Africa in 1937 and, since then, has slowly been more prevalent in both Europe and the United States [1,2].

Most patients infected with WNV remain asymptomatic (>80%). However, few develop constitutional symptoms, including fever, chills, myalgias, and vomiting. Less than 5% of patients develop neurologic involvement called WNV encephalitis. These symptoms include meningitis, encephalitis, flaccid paralysis, and myoclonus, among others [2]. Anton-Babinski syndrome, also known as Anton syndrome, is cortical blindness resulting from a lesion of the bilateral occipital lobes [3]. Here we describe a unique case of neuroinvasive WNV encephalitis from the United States that presented with fever, acute encephalopathy, and Anton syndrome. This is the first recorded association between the WNV and Anton syndrome.

## Case presentation

A 55-year-old female presented to a community hospital during the summer with a two-day history of acute onset subjective fever, myalgias, arthralgias, and altered mental status. No allergies, recent infections, or sick contacts were identified. A relative of the patient confirmed that she is a housewife and spends most of her time gardening outdoors. Vital signs on the day of presentation were significant for a fever peaking at 39.2 degree Celsius and a blood pressure of 143/86 mmHg. A comprehensive physical exam was completed significant for encephalopathy as she was oriented only to person and intermittently time. During the neurologic exam, her pupils were equal and bilaterally reactive to light with a consensual response. However, they had no response to visual threat. The patient was unable to make eye contact with hospital staff and was often seen localizing individuals based on the sound of their voices. When the patient was questioned on this, she would intermittently respond saying her vision was intact and would create visual confabulations to support that. No other focal neurologic deficits were noted on the exam. The patient required staff support with eating, drinking, and stooling.

**Figure 1:**
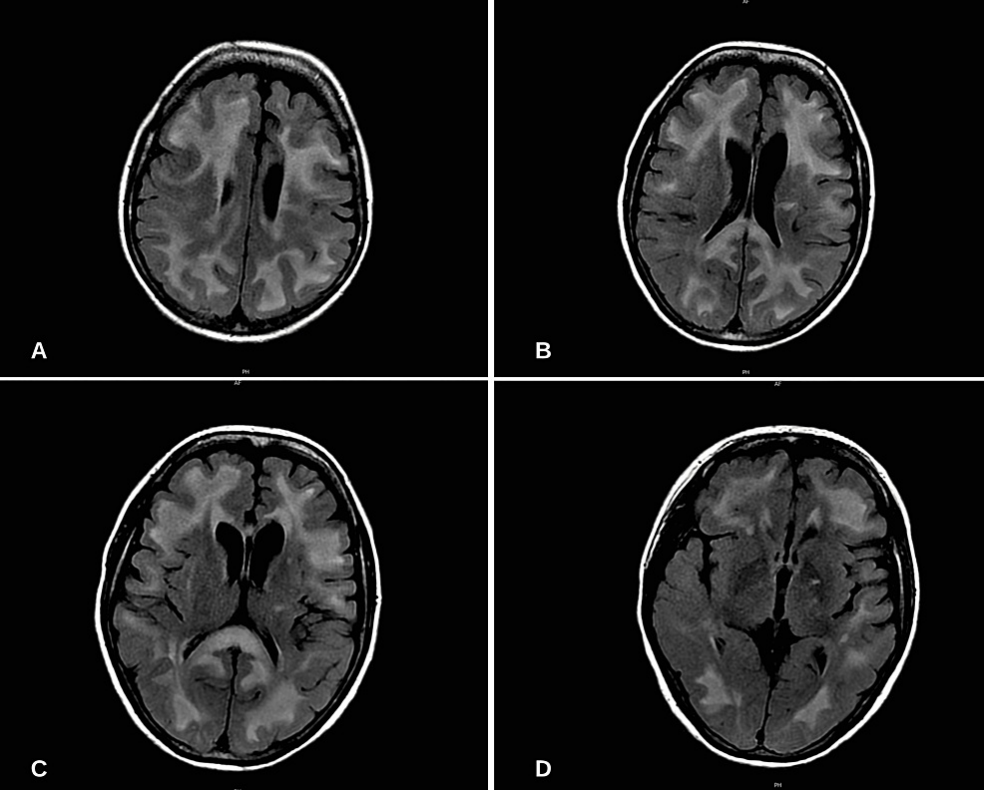
MRI brain T2-fluid attenuated inversion recovery (FLAIR) axial view obtained on patient admission A-B: significant T2-hyperintensities in both bilateral frontoparietal and occipital cortices; C-D: Significant T2-hyperintensities in subcortical structures

Initial infectious workup including blood and urine cultures were obtained that later resulted in no growth, and a chest radiograph was completed showing no acute cardiopulmonary process. A comprehensive metabolic panel (CMP), complete blood count (CBC), Vitamin B12, and thyroid-stimulating hormone (TSH) levels were obtained. Notable labs included an elevated white blood cell count of 19,000 and an absolute neutrophil count (ANC) of 8,900. A lumbar puncture and magnetic resonance imaging (MRI) of the brain with and without contrast was obtained. Imaging revealed extensive T2 hyperintensities involving the frontoparietal and occipital cortex on the T2-fluid attenuated inversion recovery (FLAIR) sequence. Mild hyperintensities were also seen in subcortical structures including the basal ganglia and thalamus (Figure 1). Cerebrospinal fluid (CSF) studies revealed elevated protein at 60 mg/dL (15-45). At this time, no neuroinfectious or autoimmune investigation was completed in the CSF.

**Figure 2:**
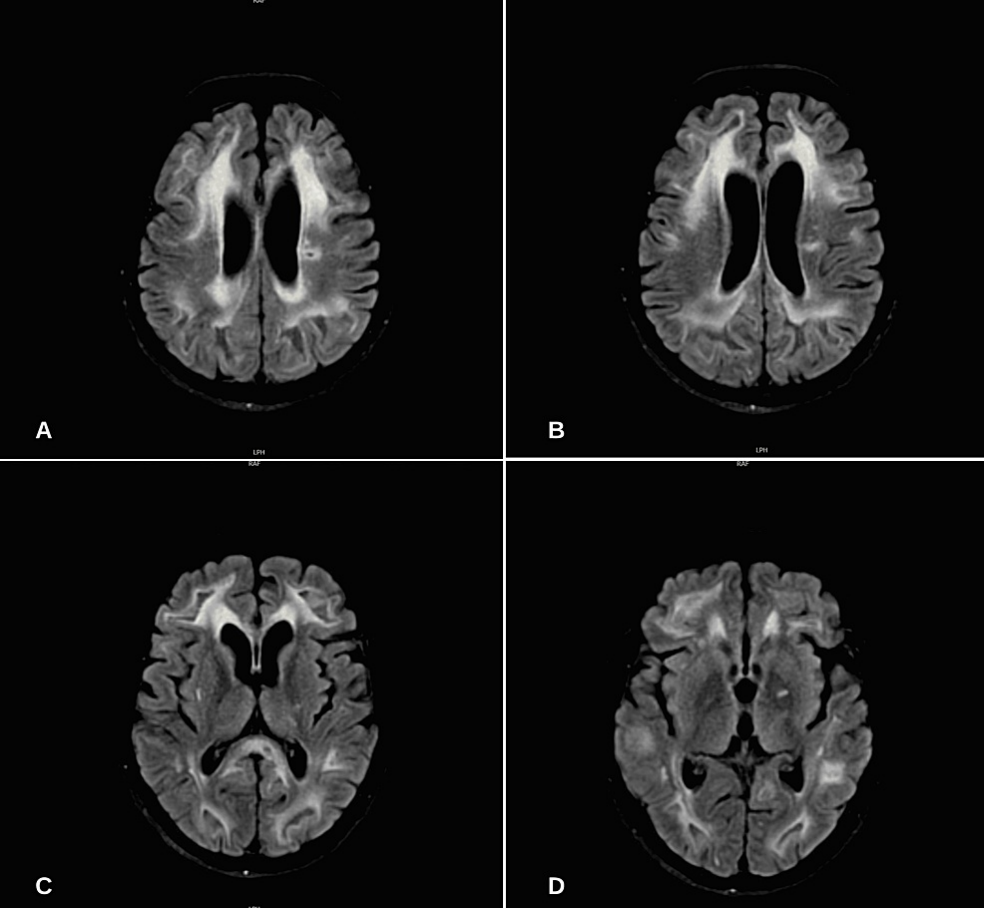
MRI brain T2-fluid attenuated inversion recovery (FLAIR) axial view obtained during week seven of the admission A-B: Chronic evolution of T2-hyperintensities in both bilateral frontoparietal and occipital cortices; C-D: Chronic evolution of T2-hyperintensities in subcortical structures

A diagnosis of posterior reversible encephalopathy syndrome (PRES) was made. Throughout admission, the patient was noted to have been normotensive, euglycemic, and without metabolic derangements. The patient was not on any immunosuppressive medications. The patient was continuously monitored and, on hospital week seven, was transferred to a tertiary care center for further neurologic evaluation due to non-improvement. A repeat MRI of the brain with and without contrast was completed, showing interval progression of the T2 hyperintensities previously noted (Figure 2).

A repeat CSF and serum evaluation was obtained, including a viral panel that showed elevated WNV Immunoglobulin G (IgG) antibodies in CSF and serum of 1.89 (normal <1.30) and 2.89 (normal <2.62) respectively as shown in Table 1. A WNV antibody neutralization assay was completed and was positive. The patient was trialed on a seven-day course of high-dose steroids with intravenous methylprednisolone 1,000 mg per day with no response. Due to a poor prognosis, the patient was referred to a long-term care facility.

**Table 1:** Cerebrospinal fluid (CSF) and serum studies obtained during the seventh week of the admission CSF: Cerebrospinal fluid; RBC: Red blood cell; WBC: White blood cell; VDRL: Venereal Disease Research Laboratory; WNV; West Nile virus; IgG: Immunoglobulin G; IgA; Immunoglobulin A; IgM; Immunoglobulin M; Ab: Antibody; DNA: Deoxyribonucleic acid; JCV: John Cunningham virus; PCR: Polymerase chain reaction; ANCA: Antineutrophil cytoplasmic antibody; ANA: Anti nuclear antibody; RT-QuIC: Real-time quaking-induced conversion;  AMPAR1: AMPAR Receptor 1; AMPAR2:  AMPAR Receptor 2; ANNA1: Type 1 antineuronal nuclear antibody; ANNA2: Type 2 antineuronal nuclear antibody; ANNA3: Type 3 antineuronal nuclear antibody; CASPR-2: Contactin-associated protein-like 2; CRMP5: Collapsin response-mediator protein-5; DPPX: Dipeptidyl peptidase-like protein 6; GABA-B: Gamma-aminobutyric acid-B; GAD65: Glutamic acid decarboxylase 65; LGI-1: Leucine-rich glioma-inactivated protein 1; NMDAR: N-methyl-D-aspartate receptor; PCA-Tr: Purkinje Cell cytoplasmic antibody type Tr; PCA1: Purkinje Cell cytoplasmic antibody type 1; VGCC: Voltage-gated calcium channel; VGKC: Voltage-gated potassium channel; RNA: ribonucleic acid

Lab Parameter	Numerical value with units and reference range
Glucose, CSF	85 mg/dL (50-80)
Protein, CSF	97 mg/dL (15-45)
RBC, CSF	3 (0)
Appearance, CSF	Clear
WBC, CSF	0
VDRL, CSF	Non-reactive
Lymphocytes %, CSF	80%
Mono/Macrophage %, CSF	20%
Total Cells Counted, CSF	5
IgG, CSF	6.2 mg/dL (0.0-6.0)
Albumin, CSF	45 mg/dL (0-35)
Albumin Nephelometry	2553 mg/dL (3500-5200)
Albumin Index	17.6 (0.0-9.0)
IgG Index	0.57 (0.28-0.66)
CSF IgG/Albumin Ratio	0.20 (0.09-0.25)
CSF Oligoclonal Bands	Negative
CSF IgG Synthesis Rate	6.8
Herpes Simplex 1 PCR, CSF	Non-reactive
Herpes Simplex 2 PCR, SF	Non-reactive
*Haemophilus Influenzae* DNA, CSF	Non-reactive
*Listeria Monocytogens* DNA, CSF	Non-reactive
*Streptococcus agalactiae* DNA, CSF	Non-reactive
*Streptococcus pneumonia* DNA, CSF	Non-reactive
Cytomegalovirus DNA, CSF	Non-reactive
Enterovirus DNA, CSF	Non-reactive
Human Herpes Virus 6 DNA, CSF	Non-reactive
Human parechoviruses DNA, CSF	Non-reactive
*Cryptococcus neoformans/gattii* DNA, CSF	Non-reactive
JCV Quantitative PCR, CSF	Non-reactive
ANCA	Negative
Mitochondrial Ab	Negative
Thyroglobulin Ab	7 (<10)
ANA Titer	<1:40 (<1:40)
Proteinase-3 Ab	< 1.0 (<1.0)
Thyroid Peroxidase Ab	1 IU/mL (<9)
14-3-3 Protein Tau, Total, CSF	Negative
RT-QuIC, CSF	Negative
WNV, IgG Ab, CSF	1.89 (<1.30)
WNV IgM Ab, CSF	1.10 (<1.30)
WNV IgG Ab, Serum	2.89 (<2.62)
WNV IgM Ab, Serum	<1.10 (<1.10)
WNV Ab Neutralization Confirmatory Test	Positive
St. Louis Encephalitis IgG Ab, CSF	<1:10 (<1:10)
St. Louis Encephalitis IgM Ab, CSF	<1:10 (<1:10)
St. Louis Encephalitis IgG Ab, Serum	<1:10 (<1:10)
St. Louis Encephalitis IgM Ab, Serum	<1:10 (<1:10)
Japanese Encephalitis IgG Ab, CSF	<1:10 (<1:10)
Japanese Encephalitis IgM Ab, CSF	<1:10 (<1:10)
Japanese Encephalitis IgG Ab, Serum	<1:10 (<1:10)
Japanese Encephalitis IgM Ab, Serum	<1:10 (<1:10)
Powassan Virus IgG Ab, CSF	<1:10 (<1:10)
Powassan Virus IgM Ab, CSF	<1:10 (<1:10)
Powassan Virus IgG Ab, Serum	<1:10 (<1:10)
Powassan Virus IgG Ab, Serum	<1:10 (<1:10)
SARS-CoV-2 RNA PCR, CSF	Negative
Vitamin A	81 mcg/dL
Acetylcholine Receptor Ganglionic Ab	<53 (<53)
Anti-SOX 1 Ab	<11 (<11)
AMPAR1 Ab	Negative
AMPAR2 Ab	Negative
Antiphysin Ab	<11 (<11)
ANNA1 Ab	<11 (<11)
ANNA2 Ab	<11 (<11)
ANNA3 Ab	Negative
Aquaporin 4 Ab	Negative
CASPR-2 Ab	Negative
CV2/CRMP5 Ab	<11 (<11)
DPPX Receptor Ab	Negative
Epstein Barr Virus DNA, Quantitative	Not detected
GABA-B Receptor Ab	<11 (<11)
GAD65 Ab	<11 (<11)
LGI -1 Ab	Negative
Ma2/Ta Ab	<11 (<11)
Myelin Ab	Negative
NMDAR Ab	Negative
PCA-Tr Ab	Negative
PCA1 Ab	<11 (<11)
VGCC Type N Ab	<55 (<55)
VGKC Protein Complex, Ab	<50 pmol/L (<50)
VGCC Protein Complex Ab	<30 pmol/L (<30)
Zinc Protein 4 Ab	<11 (<11)
Titin Ab	<11 (<11)
Phosphatidylserine IgA	<20 (<20)
Phosphatidylserine IgM	<10 (<10)
Phosphatidylserine IgG	<15 (<15)
Recoverin Ab	<11 (<11)
Striated Muscle Ab	Negative

## Discussion

**Figure 3:**
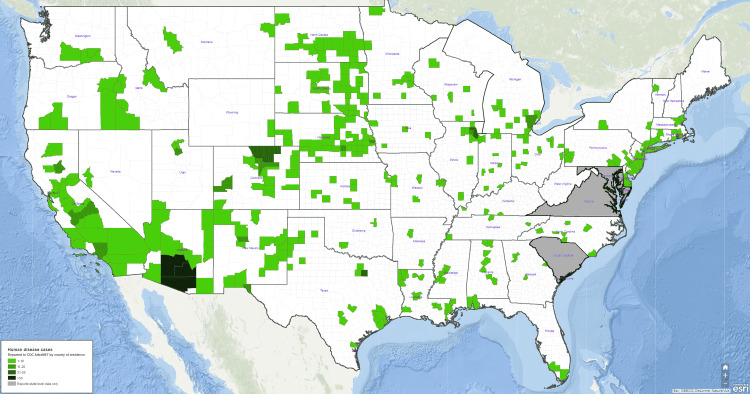
Map of the United States depicting the geographical distribution of West Nile virus (WNV) human disease cases in 2021 Source: https://wwwn.cdc.gov/arbonet/maps/ADB_Diseases_Map/index.html

The genus flavivirus is estimated to have greater than 70 viruses in total based on their genomic traits. The WNV is a flavivirus that was first described by Smithburn et al. in Uganda, Africa in 1937 and the first recorded case in the United States was in New York in 1999, known as the NY99 strain. Since then, WNV has had an increasing prevalence with outbreaks throughout Europe, Canada, and the United States. The summer of 2002 was notable in the United States for the greatest increase in the number of cases and geographic spread of WNV [4]. With the spread of WNV throughout the world, various strains with differing virulence and pathogenicity have been identified. Two main genetic lineages of the WNV are known as lineage I and II. Lineage I has been associated with cases of encephalitis throughout the world, while lineage II remains isolated from enzootic infections mainly in Africa. No cases of human encephalitis from lineage II have been identified [5]. In 2021, most human WNV cases were concentrated in the southwest portion of the United States with a small cluster of cases seen in the northeast United States (Figure 3). The epidemiological data and map depicted in Figure 3 have been provided by the Center for Disease Control (CDC) and ArboNET, the national arboviral surveillance system [6]. Reference to specific commercial products, manufacturers, companies, or trademarks does not constitute its endorsement or recommendation by the US Government, Department of Health and Human Services, or Centers for Disease Control and Prevention. The material is otherwise available on the agency website free of charge.

The WNV is a neurotropic virus, and its primary vector of transmission is the mosquito. The primary species for the transmission of WNV to humans is the Culex species. Salivary factors have been identified in mosquitoes that allow for passive replication of the virus creating a perfect breeding ground for eventual transmission. The WNV is known to cross the blood-brain barrier (BBB) through various mechanisms including passive cellular transport, axonal transport, and direct inflammation-induced breakdown of the BBB itself [7]. Our patient initially presented in the summer when mosquitoes are more active, and she was known to be an avid gardener, which presents a history consistent with her infection. We are unable to identify the exact mechanism of penetration into the BBB. However, with the profound febrile response seen (39.2 degree Celsius), the latter-most theory of inflammatory breakdown of the BBB is plausible.

After penetrating the BBB, the WNV causes direct parenchymal inflammation of the central nervous system (CNS) and, depending on the location of the lesion, varying focal neurologic deficits are seen. Table 2 summarizes the various barrier systems in the CNS [8]. The WNV that invades the CNS is termed neuroinvasive West Nile disease or encephalitis. CNS involvement from WNV infection continues to be rare, with less than 5% of reported WNV cases [2]. MRI of the brain of patients infected with WNV has shown that primarily subcortical structures are affected including the basal ganglia, thalamus, and cerebellum in a symmetric pattern. However, few cases of cortical involvement have also been identified in the mesial temporal lobe [9]. Similar neuroradiographic patterns are seen in autoimmune encephalitis [10]. Similarly, in our patient, MRI confirmed T2 hyperintensities in subcortical structures, including the basal ganglia and thalamus. Additionally, bilateral occipital lobe T2 hyperintensities were also seen which correlate with the patient’s symptoms of cortical blindness. T2 hyperintensities reflect demyelination and axonal loss.

**Table 2:** Barrier systems within the central nervous system (CNS) BBB: Blood-brain barrier; CSF: Cerebrospinal fluid

Unit	Restricting Factor	Components	Location
BBB	Endothelial cell – tight junctions	Astrocyte end-feet, pericytes, capillary basement membrane.	Cerebral blood vessels
Blood-CSF Barrier (Choroid Plexus)	Epithelial cell and tanycyte – tight junctions	Astrocyte end-feet, microglial cells	Choroid plexus and circumventricular organs
Brain-CSF Barrier (Pia)	Endothelial-cell tight junctions	Astrocyte end-feet, fenestrated dural vessels	Pia mater (pial blood vessels)
CSF-Brain Barrier (Embryonic)	Ependymal cells – strap junctions (Ventricular lining)	Radial glial cells, neuroepithelial cells	Ventricular system

**Figure 4:**
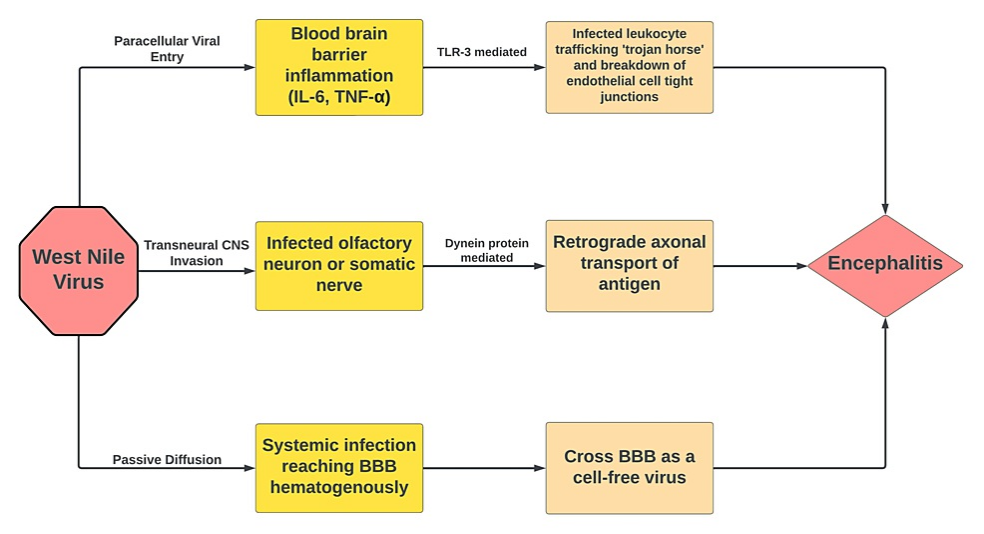
Proposed mechanisms of West Nile virus (WNV)-mediated encephalitis BBB: Blood-brain barrier; IL-6: Interleukin-6; TNF-α; Tumor necrosis factor-α; TLR-3: Toll like receptor-3

In flavivirus infections, symptoms are generally either present as a limited disease (fever and arthralgias), hemorrhagic fever, or neuroinvasive disease. Many cases of neuroinvasive West Nile disease have shown neuromuscular sequelae of symptoms that include flaccid paralysis (infected anterior horn cells), respiratory failure, and poliomyelitis-like symptoms [9,11]. Seizures presenting secondary to WNV encephalitis with an epileptogenic focus in the occipital lobe have also been recorded [12]. In severe cases of neuroinvasive West Nile disease, altered levels of consciousness lasting more than 24 hours have also been reported that are sometimes irreversible [13]. Figure 4 highlights various proposed mechanisms of WNV-mediated encephalitis.

**Figure 5:**
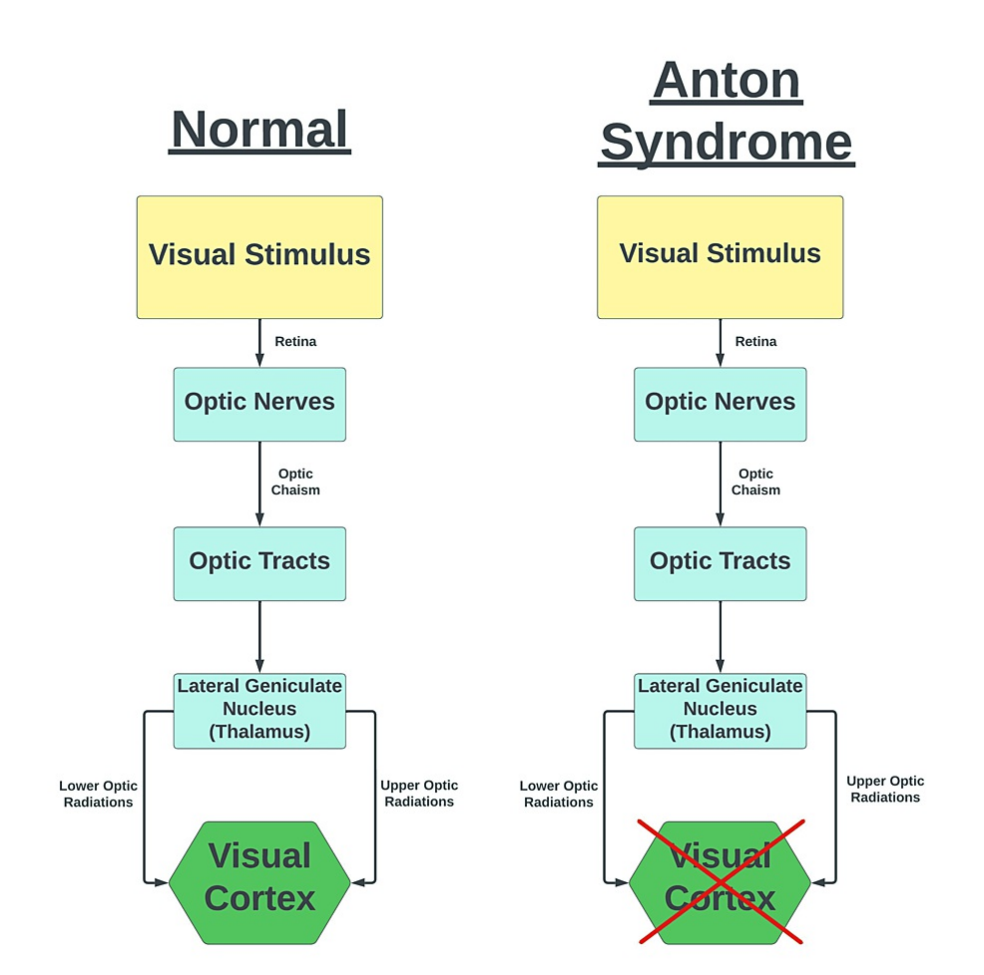
Normal visual circuitry compared to bilateral visual cortex lesion associated with Anton syndrome

Anton syndrome is visual anosognosia that occurs secondary to an occipital lobe lesion with intact anterior optic pathways. Individuals diagnosed with Anton syndrome are effectively cortically blind. However, they are unable to perceive or recognize that they are blind and produce visual confabulations such as in our patient. Many of these patients will attempt to go about their daily lives in denial of their loss of vision. Anton syndrome usually develops after injury to the bilateral occipital lobes, with the most common offender being a cerebrovascular accident (CVA) [3]. The anterior visual pathways of our patient were intact since her pupils were equal and reactive to light bilaterally, with a consensual pupillary response. However, the patient was unable to make eye contact with hospital staff and was attempting to localize individuals in the room based on auditory input. Furthermore, our patient made visual confabulations about items in the room and insisted her vision was normal. She required daily assistance with normal tasks, including eating, drinking, and stooling. Figure 5 provides a visual representation of the neural circuit affected by Anton syndrome.

Few cases of Anton syndrome have been associated with viral diseases, most notably the human immunodeficiency virus (HIV) [14]. No such cases have previously been reported secondary to the WNV or any other flavivirus. Posterior reversible encephalopathy syndrome (PRES) often occurs secondary to loss of cerebral autoregulation, notably through hypertensive episodes, metabolic derangements (i.e. hypoglycemia), or medication-induced. Our patient was not significantly hypertensive on presentation nor had significant metabolic derangements, as confirmed in the initial lab work. Medication-induced PRES often occurs secondary to immunosuppressive drugs such as tacrolimus, which our patient was not on [15]. One hypothesis is that WNV infection led to acute demyelinating encephalomyelitis (ADEM), which then may have precipitated the subsequent Anton syndrome seen in this patient. However, most ADEM cases are seen in children and show asymmetric magnetic resonance imaging findings and do not involve subcortical structures [16]. Another such hypothesis is that WNV infection may have led to a post-infectious PRES etiology. Cases of infection or sepsis-mediated PRES have been reported [17].

Diagnosis of WNV occurs through both serum and CSF analysis for immunoglobulin M (IgM) and IgG antibodies. In our case, the IgM antibodies were negative, and IgG antibodies were present in serum and CSF. Unfortunately, the initial CSF panel obtained for this patient did not contain a comprehensive infectious panel from the CSF, so the presence of WNV IgM antibodies was not tested. It was not until week seven of presentation that a repeat CSF panel and serum study was obtained that showed WNV IgG in CSF and serum. Furthermore, a virus neutralization test was performed at a state laboratory to confirm the presence of antibodies against the WNV. The exact time to the persistence of IgM antibodies is variable. Most studies have shown that IgM levels are undetectable one-three months after an acute viral infection. Fewer studies have shown the persistence of IgM antibodies one year after infection in a small subset of patients [18,19]. The lack of an initial panel of CSF and serum flavivirus is a limitation of this study. However, given the patient’s history of outdoor activities, presentation during a summer month, and symptoms on presentation including myalgias, arthralgias, and spiking fever the diagnosis of WNV is likely. Furthermore, radiographic findings that involve subcortical lesions are consistent with infection of a flavivirus. The treatment of neuroinvasive West Nile disease remains poor with no known curative or therapeutic agent. However, interferon-alpha and purified immunoglobulins with WNV antibodies may be of therapeutic potential in the future [1]. Thus, the patient was referred to a long-term care facility. Future studies are warranted on therapeutic interventions for managing WNV encephalitis.

## Conclusions

Cases of WNV infection continue to grow throughout the world and in the United States since it was first identified in 1999. Most cases of WNV are seen in the southwest United States. Neuoinvasive West Nile disease carries a catastrophic prognosis leading to irreversible neurologic deficits in many patients. We report a unique case of WNV encephalitis that presented with Anton syndrome. Anton syndrome presents with visual confabulations and occurs secondary to bilateral occipital lobe lesions. Radiographic findings included T2 hyperintensities throughout subcortical structures and the bilateral occipital lobes. Symptoms of myalgias, arthralgias, and high spiking fevers especially during the Summer months should always be concerning for WNV infection. The WNV can cross the BBB and cause encephalitis through multiple mechanisms including paracellular viral entry, trans-neural CNS invasion, and passive diffusion. Anton syndrome continues to be a rare focal neurologic deficit that has previously never been associated with the WNV or other members of the flavivirus genus.
